# Regional Differences in Dynamic Cerebral Autoregulation in the Healthy Brain Assessed by Magnetic Resonance Imaging

**DOI:** 10.1371/journal.pone.0062588

**Published:** 2013-04-30

**Authors:** Mark A. Horsfield, José L. Jara, Nazia P. Saeed, Ronney B. Panerai, Thompson G. Robinson

**Affiliations:** 1 Department of Cardiovascular Sciences, University of Leicester, Leicester, United Kingdom; 2 Departamento de Ingeniería Informática, Universidad de Santiago de Chile, Santiago, Chile; 3 NIHR Biomedical Research Unit for Cardiovascular Sciences, The Glenfield Hospital, Leicester, United Kingdom; Charité Universitaetsmedizin Berlin, Germany

## Abstract

A novel method is described for mapping dynamic cerebral blood flow autoregulation to assess autoregulatory efficiency throughout the brain, using magnetic resonance imaging (MRI). Global abnormalities in autoregulation occur in clinical conditions, including stroke and head injury, and are of prognostic significance. However, there is limited information about regional variations. A gradient-echo echo-planar pulse sequence was used to scan the brains of healthy subjects at a rate of 1 scan/second during a transient decrease in arterial blood pressure provoked by a sudden release of pressure in bilateral inflated thigh cuffs. The signal decrease and subsequent recovery were analyzed to provide an index of autoregulatory efficiency (MRARI). MRI time-series were successfully acquired and analyzed in eleven subjects. Autoregulatory efficiency was not uniform throughout the brain: white matter exhibited faster recovery than gray (MRARI = 0.702 *vs.* 0.672, p = 0.009) and the cerebral cortex exhibited faster recovery than the cerebellum (MRARI = 0.669 *vs.* 0.645, p = 0.016). However, there was no evidence for differences between different cortical regions. Differences in autoregulatory efficiency between white matter, gray matter and the cerebellum may be a result of differences in vessel density and vasodilation. The techniques described may have practical importance in detecting regional changes in autoregulation consequent to disease.

## Introduction

Cerebral autoregulation (CA) describes the mechanism responsible for maintaining cerebral blood flow (CBF) relatively constant when there are changes in mean arterial blood pressure (ABP) [1]. Abnormalities in autoregulation have been reported in a number of clinical conditions, including stroke, orthostatic hypotension, carotid artery disease, and head injury, and are of prognostic significance [2]. Two CA regimes are defined: ‘static’, referring to the steady-state cerebrovascular response to long-term changes in blood pressure, and ‘dynamic’, in response to sudden fluctuations in perfusion pressure. The introduction of transcranial Doppler (TCD) ultrasound made it possible to measure instantaneous changes in the CBF velocity in the left and right middle cerebral arteries, and enabled development of a method for assessing dynamic CA (dCA) [3]. This has now become the standard way to estimate dCA in humans, and has largely replaced measurements of static CA [4], [5]. Relatively fast changes in mean ABP can be induced by a number of different maneuvers, such as the Valsalva maneuver, hand-grip, cold stress test or changes in posture [6], [7].

One of the most commonly used is the sudden release of inflated thigh cuffs, the original approach proposed by Aaslid *et al.* [4], which causes a sudden drop in mean ABP. The drop and subsequent recovery of blood flow velocity are usually quantified using an autoregulatory index (ARI), which is obtained by fitting one of 10 possible CBF velocity template response curves, each corresponding to an ARI value ranging from 0 (absence of autoregulation) to 9 (best observed autoregulation). Subsequent work has shown that ARI values can also be extracted by modeling the dynamic relationship between mean ABP and CBF velocity resulting from spontaneous fluctuations in these variables [8].

TCD-derived ARI and other dCA parameters have been shown to detect pathological changes in CA, but wider clinical application has been hindered by the poor spatial resolution of TCD, which only allows detection of differences between the cerebral hemispheres. Greater spatial discrimination in CA assessment would be highly desirable in many clinical situations, such as the evaluation of focal ischemia and the surrounding tissue in stroke patients and those with posterior reversible encephalopathy syndrome [9], as well as regional differences in traumatic brain injury and migraine [10]. We have previously shown that magnetic resonance imaging (MRI) can be used to assess autoregulatory efficiency, providing greater consistency for both inter-hemisphere comparisons and repeated measures than the TCD technique [11]. We report here on a re-analysis of the data presented in [11] (supplemented by two additional subjects), using a novel image analysis method to assess the MRI data acquired to assess regional dCA. We used the thigh-cuff maneuver to induce ABP perturbations, since this causes a rapid ABP change, and can be easily applied within the confines of an MRI scanner. In this pilot study, we have used this method to test whether there is evidence for regional and tissue-specific differences in autoregulation, in a small group of healthy control subjects. This adds to the previously-published study [11] by showing for the first time that maps of autoregulatory efficiency can be produced, with an analysis of the regional variation in autoregulation that goes beyond simple between-hemisphere differences.

## Materials and Methods

### Subjects

Twelve healthy subjects (8 male) of mean age 58±13 years (range 41–75) were recruited from departmental volunteers and responses to advertisements placed in local health clubs. Subjects were excluded if they had a history of cardiovascular and stroke disease, migraine, epilepsy, or other chronic neurological disorders, or if they had contraindications to MRI scanning. The study was approved by the Leicestershire, Northamptonshire and Rutland Research Ethics Committee (REC 09/H0403/25), and all subjects gave written informed consent.

### Transcranial Doppler Ultrasound

Prior to MRI scanning, all subjects underwent a TCD assessment of autoregulation, with ABP perturbation brought about by rapid deflation of bilateral thigh cuffs, as previously described and reported [11]. During cuff deflation, ABP was measured non-invasively using the finger arterial volume clamping technique (Finapres, Ohmeda 2300, Louisville, CO, USA), with the BP cuff applied to the middle phalanx of the middle finger of one hand. This allowed us to verify that a rapid ABP fall was reliably achieved on release of the bilateral thigh cuffs.

### MRI Scanning

All MRI measurements were performed using a 1.5 Tesla MRI scanner (Symphony, Siemens AG, Germany) with a transmit/receive single channel quadrature birdcage head coil. After arrival at the MRI suite and being seated for a period of 10 minutes, brachial ABP was measured using a validated cuff device (Model UA 767, Omron Healthcare Co. Ltd, UK) as a guide for thigh cuff inflation pressures. Subjects then lay supine on the scanner couch and large inflatable cuffs (Model C22, Hokanson Inc., USA) were wrapped around each thigh. A lightweight foam collar was used to support the head and neck, and a custom-made foot rest was clamped to the couch to provide additional support during the cuff deflation maneuver. This is a wooden plate that projects upwards from the couch and which is adjustable to provide a firm base against which the subjects could brace their feet at a comfortable ankle flexion angle.

After a localizer scan, standard proton-density/T_2_-weighted dual-echo fast spin-echo and spin-echo echo-planar imaging (EPI) diffusion-weighted (DW) sequences were performed so that any existing pathology could be identified. The sequence parameters for the dual-echo scan were: TR = 2930 ms; TE = 15,86 ms; echo train length = 5; matrix = 256×256; in-plane resolution = 0.9×0.9 mm. For the DW sequence, the parameters were: TR = 4100 ms; TE = 112 ms; matrix = 128×128; in-plane resolution = 1.8×1.8 mm interpolated to 0.9×0.9 mm. Diffusion weighting was applied in three orthogonal directions, with a *b*-factor of 1000 s mm^−2^; an image with *b* = 0 was also collected. For both sequences, 21 axial slices were acquired, with a slice thickness of 5 mm and a 2 mm gap between slices, covering the whole of the cerebrum. Slices were positioned parallel to a line that joined the most anterio-inferior and posterio-inferior margins of the corpus callosum as viewed on a sagittal localizer, correcting for any head tilt by also viewing in the coronal plane.

The thigh cuffs were then inflated to at least 20 mmHg above peak systolic BP and the main image series was commenced as soon as the cuffs were up to pressure. This consisted of rapid serial acquisition using a gradient-echo EPI sequence (TR = 1000 ms; TE = 40 ms; flip angle = 40°; matrix = 64×64; in-plane resolution = 3.45×3.45 mm; slice thickness = 5 mm; gap between slices = 4 mm). Twelve slices were positioned as for the dual-echo and DW scans, but there was less coverage of the cerebrum due to the smaller number of slices acquired. Two hundred and forty multi-slice image sets were acquired over four minutes, equivalent to a sampling frequency of 1 Hz, which was the fastest rate possible while still maintaining coverage of the head. The relatively low resolution of the image matrix (4096 voxels per slice) was also because of the need for high sampling frequency. Three minutes into the series the cuffs were rapidly deflated. It has previously been found to be necessary to inflate the thigh cuffs for three minutes prior to deflation, in order to obtain a reliable ABP drop [4]–[6], [11]. Although we ultimately only characterize the MRI signal change after deflation in order to assess autoregulation, it is advantageous to scan for the full four minutes to minimize any change in stress to the subject (due to the acoustic noise during MRI scanning) around the time of the thigh cuff release. This also allows us to assess and correct any signal changes due to physiological ‘noise’, as done for similar scanning protocols such as functional MRI (fMRI) analysis [12]. After the scan completed, the cuffs were re-inflated and the procedure was repeated twice more. The scanning for each subject was carried out in a single session, during which the volunteer remained supine without being removed from the scanner.

### Data Analysis

Image pre-processing was performed using the FMRIB Software Library (FSL) Version 4.1.7 (www.fmrib.ox.ac.uk/fsl). This consisted of: noise reduction using an anisotropic spatial filter; correction for subject motion during the acquisition; and registration to a standard anatomical space in order to allow data averaging across subjects. Specifically, we used the ICBM152 atlas from the McConnell Brain Imaging Centre, Montreal Neurological Institute, Canada (www.bic.mni.mcgill.ca/), with the resolution reduced to 99×117×39 voxels (approximately 2 mm×2 mm×5 mm) to save computation time during image processing. Registration to the atlas was performed using the proton-density weighted images, and then the same transform was applied to the low-resolution time-series of images.

The time-series of images for each subject and each repeat was then analyzed with computation of the autoregulation parameters for every voxel of the image (a voxel-by-voxel calculation) to evaluate the signal drop and subsequent recovery associated with the thigh-cuff release. First, mean signal intensity and linear drift were removed, and a high-pass filter applied (0.0125 Hz cut-off) to remove any variation due to scanner drift and physiological noise. A correction for the slight difference in time of acquisition for each image slice was also applied by interpolating the time series and resampling at a common acquisition time for all slices. The signal for each voxel was then normalized by dividing by the mean of the original signal intensities for the time-series in that voxel. In analogy with the standard method for assessing autoregulatory efficiency using TCD ultrasound [4], the signal drop and recovery in response to the thigh-cuff maneuver were analyzed by fitting a function that can vary in the time required to return to baseline after perturbation, and which can also overshoot the baseline if the recovery is rapid. However, this differs from the ultrasound-based ARI since we used a function of a continuous variable related to autoregulation, rather than fitting one of ten ‘template’ functions giving ordinal values from 0 to 9, as is standard practice for ARI measured using TCD.

**Figure 1:**
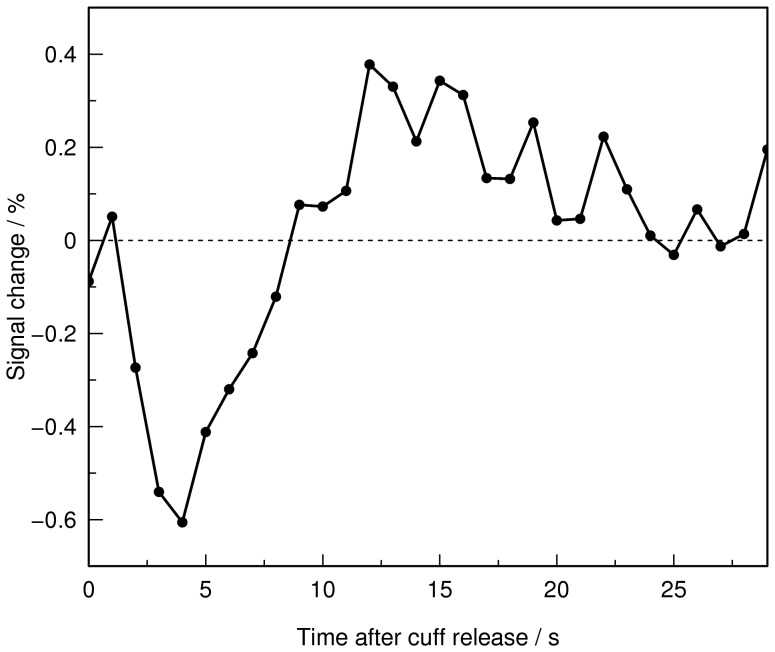
Whole brain response to the thigh cuff release. The signal intensity for the whole brain in response to the thigh-cuff release is used to identify visually the time at which the minimum intensity occurs. This figure shows the signal intensity time-course for a single thigh cuff release maneuver in one subject.

**Figure 2:**
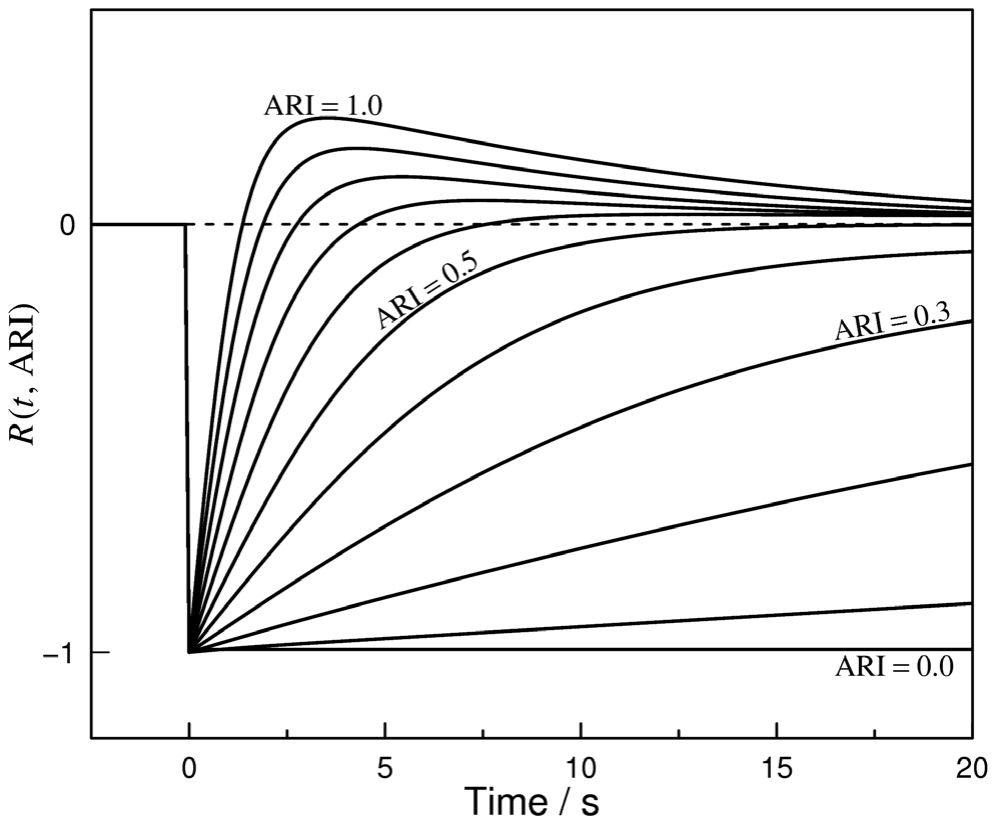
Family of autoregulation index (MRARI) curves. These curves were generated by Eq. 1, and show the range of shapes that can be adopted for different values of the MRARI parameter (for unit amplitude, *A*). Least-squares fitting is used to find the MRARI and *A* values such that the observed signal intensity data in each image voxel are best matched by the fitted curve, thus characterizing, in that voxel, the amplitude of the perturbation and speed of return of the MRI signal intensity after the thigh cuff release. The origin for the time axis is identified visually by examining the average time course for the whole brain. Eleven example curves are shown, but in the fitting procedure, both MRARI and *A* are estimated on a continuous scale.

For the whole brain, the time point at which the maximum signal intensity drop occurred (*t*_0_) was identified visually for each subject and each thigh-cuff maneuver (Figure 1). Then, for each voxel, an index of autoregulation (MRARI) was calculated by non-linear least-squares fitting of a function that models the return of the intensity to baseline after the initial drop. This function was derived empirically to give a family of curves that matched the observed time course seen for the whole brain responses of individual subjects. The return, *R*(*t*), was the sum of a hyperbolic recovery term *r*(*t*, MRARI) and an asymptotic decay term *d*(*t*, MRARI). This function monotonically approaches baseline when the MRARI <0.5, and produces an overshoot for values >0.5. The expression used was:
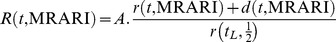
(1)in which



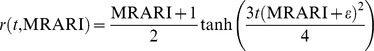
and



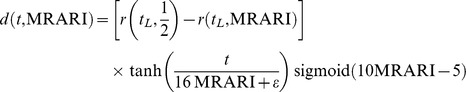
where *t* is the time elapsed since *t*_0_, *t_L_* is a time point long after *t*_0_ when the recovery is very close to its asymptote, and ε = 1/*t_L_* is a small value to guarantee the recovery of the signal and the decay of the overshoot in the long term. The two fitted parameters are the amplitude, *A*, of the signal drop following thigh-cuff release and the MRI-derived autoregulation index, MRARI. Constants were derived empirically to match observed signal recovery curves. The value of *t_L_* used was 10^3^ s. A set of curves produced by *R*(*t*) for unit amplitude *A*, and a range of MRARI values between 0 and 1 is shown in Figure 2. The minimum in signal intensity and 20 time points after the minimum were used in the fitting procedure (21 time points in total). When fitting this expression to individual voxels, where either the initial signal initially rose rather than dropped, or the value of the fitted MRARI parameter was outside the range [0, 1.5], this was deemed to be non-physiological, and indicated as a fitting failure. In addition, when the distribution of MRARI values across all brain voxels for one run was dominated by either low or high values (the difference between the mean and the median of their distribution was greater than a quarter of their standard deviation), the MR image time-series was considered as unreliable and the data from that run were discarded.

Maps of the amplitude of drop *A* and MRARI were calculated for each subject by averaging the fitted values across the different runs. When averaging, maneuvers with a fitting failure in a particular voxel were not included in the average for that voxel. Regional differences were examined by calculating mean signal drop and mean MRARI on the different regions of interest for every subject. Region masks were obtained from the ICBM-152 NL 2009a atlas (whole brain, white matter excluding the cerebellum, gray matter excluding the cerebellum) and the MNI-152 standard distributed with FSL (cerebellum, frontal cortex, occipital cortex, parietal cortex, and temporal cortex) [13] and then registered to the standard space in use (99×117×39 voxels) using nearest neighboring interpolation. Masks were obtained considering a threshold of 0.5 for the respective region probability maps, and removing voxels whose probability of being cerebrospinal fluid (CSF) was 0.25 or greater. This lower threshold for CSF was used because it afforded a conservative approach and led to signal contamination from CSF in the sulci of the brain being much less likely. With the exception of the whole brain, the masks do not have overlapping voxels.

### Statistical Analysis

Paired Student’s t-tests were used to compare both mean amplitude of drop and mean MRARI between two different regions of interest (ROIs). We also report Cohen's *d* for the paired Student’s t-tests as a measure of the effect size. One-way repeated-measures ANOVA was used to assess differences in both mean signal drop amplitude and mean MRARI when comparing more than two ROIs. The relationship between the amplitude of signal drop and the MRARI parameter was tested by linear regression analysis for each separate run, using the same voxels accepted for the image analysis. p<0.05 indicated a significant difference.

## Results

During the TCD protocol (pre-MRI), all subjects showed a distinct drop in mean ABP in response to the sudden release of the inflated thigh cuffs. Careful visual inspection of individual recordings led to rejection of 4 TCD recordings (out of a total of 30) due to an insufficient drop in the response profile of the ABP for TCD measurements. For the accepted TCD recordings, the mean ABP drop was 19%±11% of mean baseline values, ranging from 9.3% to 59.2%.

**Figure 3:**
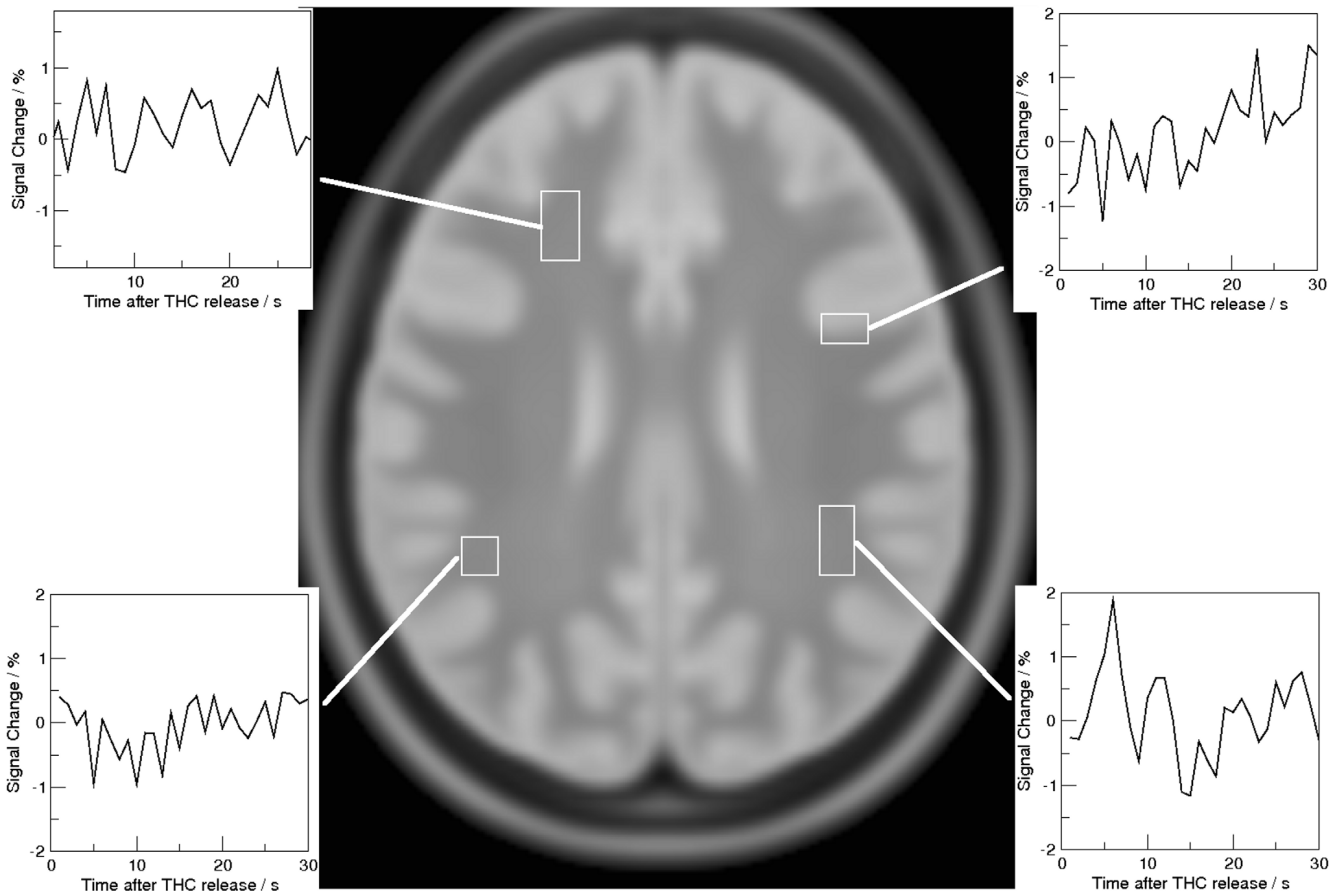
Example ‘rejected’ signal responses shown for one slice of one subject’s run. The central image is the corresponding slice from the brain atlas. The four individual regions surround groups of pixels within which all signal responses were rejected for that run, while the corresponding four graphs show the average signal response for those pixels.

**Figure 4:**
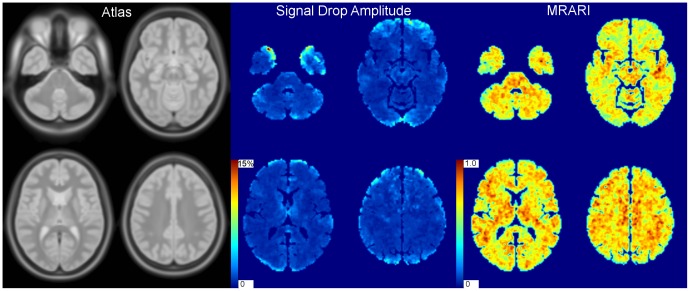
Computed autoregulation parameter maps. Four representative axial slices from the MRI brain atlas (ICBM152) are shown on the left, together with the corresponding four slices from the population averaged signal drop amplitude (center) and MRI-derived autoregulation index (MRARI) images (right).

Of the twelve subjects who underwent MRI, one withdrew from the study due to intolerance to the thigh-cuff inflation. None of the subjects had any radiological evidence of neurological abnormalities. From the remaining eleven subjects, two time series were rejected (both from the same subject), and were not included in the analysis because of excessive head motion during the four minutes of scanning. Thirty one MRI time series were successfully analyzed, with maps of the amplitude of signal drop and MRARI being constructed in standard anatomical space. For these 31 time series, the mean maximum displacement detected during motion correction was 0.79 mm ±0.70 mm. During the 10 second period starting at thigh-cuff release, where motion may be expected to be greatest, the mean maximum displacement detected was 0.41 mm ±0.37 mm. When fitting Eq. 1 voxel-by-voxel to the time series for all voxels within the brain mask (including those voxels within CSF), MRARI and amplitude values were successfully obtained for 63.7% of all voxels. Of the 36.3% of voxels where values could not be obtained, 31.8% was contributed by voxels that did not show a drop in signal at the time the mean (whole brain) signal did, and 4.50% gave MRARI outside the "physiological" range we set (less than zero or greater than 1.5). Excluded voxels were present throughout the brain, with no apparent difference between white matter (37.6% excluded) and gray matter (37.0% excluded). However, more detailed analysis showed 39.9% excluded in the frontal GM, 38.1% in the temporal, 33.6% in the parietal, 31.8% in the occipital and 31.6% in the cerebellum. Examples of excluded time series are shown in Figure 3 for one run of one subject. The parameter maps were then averaged to produce population mean amplitude and MRARI images as shown in Figure 4.

As expected when cerebral pathology is absent, comparisons between left and right brain hemispheres showed no significant differences in the mean amplitude of signal drop (1.58%±0.66% *vs.* 1.49%±0.57%; p = 0.251, d = 0.37) or MRARI (0.686±0.054 *vs.* 0.684±0.077; p = 0.877, d = 0.05). However, there was a greater signal drop in gray matter (1.81%±0.67%) than in white matter (1.18%±0.59%; p<0.001, d = 1.75), while white matter exhibited a significantly faster recovery (MRARI = 0.702±0.067) than gray matter (MRARI = 0.672±0.066; p = 0.009, d = 0.97).

We compared the frontal, occipital, parietal and temporal cortices using one-way repeated-measures ANOVA, where there were no significant differences either in the mean signal drop amplitude or the mean MRARI (see Table 1). Finally, we compared the cerebellum to a single region consisting of all four cortical regions combined; there was a greater signal drop in the cortices (1.87%±0.72%) than in the cerebellum (1.55%±0.65%; p = 0.003, d = 0.95), while the cortices exhibited a significantly faster recovery (MRARI = 0.669±0.063) than the cerebellum (MRARI = 0.645±0.064; p = 0.016, d = 0.87).

**Table 1:** Population mean ± standard deviation signal amplitude drop and MRARI values in different brain regions.

Region	Signal Drop Amplitude	MRARI
Left Hemisphere	1.58%±0.66%	0.686±0.054
Right Hemisphere	1.49%±0.57%	0.684±0.077
Gray Matter	1.81%±0.67%	0.672±0.066
White Matter	1.18%±0.59%	0.702±0.067
Frontal Cortex	2.09%±1.30%	0.672±0.055
Occipital Cortex	1.82%±0.51%	0.664±0.073
Parietal Cortex	1.61%±0.41%	0.678±0.073
Temporal Cortex	1.86%±0.82%	0.659±0.078
All cortical regions	1.87%±0.73%	0.669±0.063
Cerebellum	1.55%±0.66%	0.645±0.064

Linear regression analysis of the relationship between the amplitude of signal drop and the MRARI parameter indicated significant correlations in 27/31 runs with a positive slope in 13/27 runs and negative slope in the remainder 14/27 runs. This analysis was based on 54,128±6,717 voxels per run and the squared correlation coefficients were 0.0058±0.0063. The inconsistent distribution of slopes and the low correlation coefficients suggest that overall, MRARI was independent of the amplitude of signal drop.

## Discussion

The significant differences between gray and white matter, and between the cerebellum and cortical regions, are the first demonstration that dCA is not uniform over the whole brain in the absence of disease. This ability to map regional differences in dCA opens up a new avenue of research into human cerebrovascular function, with considerable potential to improve our understanding and clinical management of stroke and other encephalopathies. Though regional differences in CO_2_ cerebrovascular reactivity have been reported previously [14] and although mapping the response to hypercapnia is technically more straightforward, cerebrovascular reactivity does not share the same physiological pathways as CA. This explains the poor agreement between these two approaches to the assessment of vascular pathology in classical, whole-brain clinical studies [15].

The agreement between MRI and TCD responses to the thigh cuff maneuver has previously been reported [11], with Doppler ultrasound being the standard way to assess CBF changes in dCA measurement. In that study, on a large subset of the subjects studied here, there was an average reduction in CBF through the MCAs of 24% (assuming a constant MCA diameter), and a corresponding reduction in signal intensity in the MRI scans of approximately 0.5% on average, in the territories supplied by the MCAs. This is commensurate with the low capillary blood volume in brain parenchyma (3.8%–4.7% of parenchyma volume in the gray matter perfused by the MCA and 2.7% in white matter [16]). Nevertheless, we have so far not characterized the physiological responses that are behind the observed changes in MRI signal intensity, which could come from an increase in the deoxyhemoglobin concentration (as a result of reduced perfusion or increased oxygen consumption), a decrease in the blood volume or perhaps changes in blood flow. Use of both gradient-echo (as used here) and spin-echo EPI pulse sequences would allow us to assess the relative impacts of autoregulation in the large vessels versus the capillary bed [17]. Measurement of the *T*_2_* relaxation time of the blood in the sagittal sinus is sensitive to the oxygenation state of the venous blood, so allowing an assessment of whether the change in blood oxygenation in the venules is an important contributor to the signal response we see [18]. However, all such measurements would need to be made with sufficiently high temporal resolution that the dynamic response to the blood pressure perturbation can be captured.

One important feature of our approach to the quantification of intra-subject regional differences in dCA, is the use of a temporal parameter (MRARI) to express the rate of return following the signal drop induced by the sudden release of inflated thigh cuffs. Parameters that express temporal patterns of physiological signals tend to be much more robust than measures based on signal amplitude, such as measures of gain in dCA, or absolute CBF changes in static autoregulation [8]. This is an important consideration in MRI studies due to signal drift, subject motion and on-going discussions about the precise interpretation of changes in signal intensity seen in fMRI studies using the BOLD technique [19]. The statistically significant differences between the amplitude of signal drop and MRARI parameter in gray and white matter, which are borne out by the spatial distributions in Figure 4, suggest differences in autoregulatory capacity between gray and white matter that have not been reported previously. However, this difference is not entirely surprising given the greater metabolic needs of gray over white matter. The dominant view is that greater capillary density is found in gray compared to white matter [20]. As a consequence, arterioles feeding gray matter capillaries need to accommodate relatively greater blood flow, therefore requiring larger vessel diameters than corresponding arterioles perfusing white matter. This difference in micro vessel diameter might be the simplest explanation for the differences in MRARI, given many previous observations of the association between vasodilation and worsening of CA. There may also be a significant contribution to the signal change from larger arteries, such as the sub-pial perforating arteries, which could have given rise to this gray/white matter difference. From this perspective, the differences in MRARI between gray and white matter, together with the lack of right-left differences, provide evidence of the reliability of our approach, rather than entirely new physiological knowledge.

Although gray matter showed a greater drop in signal amplitude, compared to white matter (Table 1), the regression analysis between the amplitude of the drop and the MRARI parameter demonstrated that these two quantities were not linked. The fact that most of the runs analyzed demonstrated a significant correlation is not surprising given the very high number of degrees of freedom involved, represented by the number of voxels that contributed to each linear regression. Nevertheless, the sign of the slope was evenly distributed between runs and the mean correlation coefficient was extremely low thus showing a lack of consistent association.

On the other hand, differences between the cerebellum and the cortical regions are more intriguing. A previous study of autoregulation in migraine with aura sufferers reported impaired static autoregulation in both the cerebellar and anterior circulation. It was hypothesized that the cerebellar predilection of ischemic lesions in migraine with aura may be a combination of altered autoregulation and additional factors, including end artery cerebellar angio-architecture [10]. Conversely, others have shown the superior ability of the cerebellum to respond to vasodilatory stimuli. In a study using positron emission tomography [14], CBF change in response to hypercapnia was 17.5%±21.9% per mmHg in the cerebellum, while the corresponding values for the frontal cortex was 9.3%±8.8% per mmHg. Considering only gray matter, Binks and colleagues also reported larger blood flow changes in the cerebellum in response to hypoxia, in comparison to the frontal area [21]. After examining 50 different regions of interest, these authors observed that the response to hypoxia was greater in phylogenetically older regions of the brain compared to evolutionary younger regions. Such regional differences are worthy of further investigation, and indeed may have additional importance in diseased states.

A recent meta-analysis reported that CA is impaired following acute stroke, particularly in moderate-to-severe cases, though improvement is normally seen by 3 months post stroke. Most studies have assessed changes at a gross, usually hemispheral level, and have reported impaired CA in the affected hemisphere, though the unaffected hemisphere may also be involved, especially in lacunar stroke [22]. It is possible that focal regions of moderately or severely impaired CA are being masked by large areas of relatively normal CA, and that imaging of stroke patients using the new method presented here would reveal much greater CA impairment in the affected areas.

Although good-quality measurements were obtained in most subjects, the present study had some limitations. First, the signal change observed with MRI was small, and therefore it might be expected that the images would be sensitive to subject motion, particularly during cuff deflation. However, this did not appear to affect our results greatly, with only two runs from one subject being excluded. The problem of motion was, at least in part, mitigated by the use of a neck collar and a foot support, to prevent unwanted change in position or angulation of the head. Slight subject motion was also corrected during image pre-processing. Nevertheless, there are areas of large signal change on the population-averaged map in Figure 4, particularly in the occipital and frontal lobes. These larger changes are most likely the result of residual subject motion, or signal bleed from large vessels such as the sagittal sinuses. Furthermore, the images are of low spatial resolution (3.45×3.45 mm in-plane resolution, with a 5 mm slice thickness and a 4 mm inter-slice gap) and subject to further spatial averaging during image processing; this would lead to signal bleed from the CSF particularly into the cortex, although we attempted to minimize this by conservatively masking the signal from CSF. There would also have been considerable blurring of the gray-white matter boundary, which is likely to reduce the significance of any differences between gray and white matter that we observed. We were constrained in the spatial resolution achievable by the need to obtain temporal resolution good enough to measure the signal recovery after disturbance. Furthermore, we wanted to cover the whole of the cerebrum, and therefore chose to introduce a 4 mm inter-slice gap with consequently less than ideal spatial sampling, However, the use of high-field MRI scanners (3 Tesla and beyond) is likely to give both improved signal-to-noise ratio and greater signal change due to the BOLD effect [23], and permit higher spatial resolution in future studies.

Due to the long echo train, the EPI pulse sequence gives rise to considerable spatial distortions, particularly in regions of large variations in magnetic susceptibility (e.g., in the frontal lobes and the posterior fossa). This causes difficulties when registering images to an atlas, since there is sometimes a poor correspondence between tissues. This may have been the cause of some of the high signal changes seen in the population average map, in the temporal, occipital and frontal lobes seen in Figure 4. Registration to a standard atlas is also the cause of spatial blurring, that would give rise to a poor delineation of image features in atlas space, such as the boundary between gray and white matter [24]. We also observed a ‘non-physiological’ signal response in approximately 36% of all brain voxels, with a higher proportion in the frontal and temporal lobes in comparison to the parietal and occipital lobes and the cerebellum. This may also indicate the fact that there is loss of signal when using the EPI pulse sequence, particularly in the fontal and temporal lobes, due to magnetic susceptibility variations, which would give rise to poorer signal-to-noise ratio.

The main advantages and limitations of the thigh cuff maneuver we used have been previously described [5], [6], [8]. The inflation of thigh cuffs to 20 mmHg above systolic ABP, before rapid deflation, causes moderate pain. Despite this, the technique is still widely undertaken on patients [2]. Only one subject withdrew from our study, but even this was unusual in our wider experience with this technique. A further confound could arise because our setup can be viewed as a single-event fMRI experiment, with the thigh cuff release giving rise to a change in the pain stimulus caused by the application of the thigh cuff. We aimed to reproduce the conditions that occur during the normal thigh-cuff maneuver assessed using TCD, and the arguments about changes to pain status brought about by the thigh-cuff release also apply to the TCD assessment of ARI. With an imaging assessment of autoregulation, we have the advantage that if there are localized changes in blood flow then they should be visible in the centers associated with pain, such as the somatic and insular regions, the anterior cingulate gyrus and perhaps the thalamus [25]. No such changes were seen by visual inspection of the autoregulation parameter maps, although this aspect warrants more detailed investigation when more sophisticated protocols become available.

Finally, because of a lack of MRI-compatible blood pressure measurement devices, unfortunately the ABP stimulus could not be measured in the MRI scanner to confirm that a rapid drop in ABP had been achieved. However, a previous analysis showed that the patterns of CBF velocity measured with TCD and the MRI signal intensity following thigh cuff release are highly correlated, and that ABP has only a small coefficient of variation in serial measurements, implying that ABP drop during MRI scanning was very similar to that recorded during the TCD examination [11].

In summary, MRI-based assessment provides information about the spatial CA variation that is not available from the standard TCD-based approach. This has great potential to guide clinical ABP management in diseases such as stroke and traumatic brain injury.
